# The Association of Physical and Mental Illness and Self-Harm Resulting in Hospitalization: A Population-Based Study of Older Adults in South Korea

**DOI:** 10.3390/ijerph19148303

**Published:** 2022-07-07

**Authors:** Sangmi Kim, Haesang Jeon, Joonhyeog Park

**Affiliations:** 1 Department of Health Management, Jeonju University, Jeonju-si 55069, Korea; seasea12@jj.ac.kr; 2 Department of Social Welfare, Jeonju University, Jeonju-si 55069, Korea; 3 Department of Social Welfare, Seoul National University, Seoul 08826, Korea; pjoon94@snu.ac.kr

**Keywords:** self-harm, older adult, Korean, physical disease, mental disorder

## Abstract

Self-harm injury among older adults is a pressing problem that demands social attention in South Korea. This study sought to identify the association between physical and mental illness and hospitalization following self-harm injuries, compared to non-self-harm injuries, among older adults living in Korea. We analyzed individuals aged 65 and older who were admitted to hospitals either for self-harm or non-self-harm from a population-based survey of the Korea National Hospital Discharge In-depth Injury Survey (KNHDIS). A logistic regression analysis was performed. Compared with non-self-harm-related hospitalization, self-harm hospitalization was associated with higher odds of depression, other disorders of the nervous system, malignancies, alcohol misuse and dependence, and drug-related dependence. Dementia, anxiety disorder, diabetes, arthritis, cerebral palsy, and other paralytic syndromes had a lower likelihood of leading to self-harm than non-self-harm hospitalization. The findings of this study can inform medical professionals to identify older adults with a heightened risk of self-harming behavior leading to hospitalization.

## 1. Introduction

Deliberate self-harm is a serious public health problem in that the attempt to die by suicide and subsequent hospitalization not only cause suffering to families, caregivers, and health professionals, but also incur high social costs [1,2,3,4]. *The World Health Organization* (WHO) reports that approximately 800,000 people die by suicide every year, with as many as 16 million people attempting suicide. As of 2019, suicide was ranked as the 20th highest cause of death—higher than malaria, HIV, breast cancer, and war [5]. In particular, South Korea (hereafter referred to as Korea) has the highest suicide rate in the world, almost double the OECD average [6], and is characterized by a very high suicide rate in older adults. According to a recent report from the *Korea Foundation for Suicide Prevention*, the suicide rate per 100,000 people in Korea is 32.9 for those in their 60s, 48.9 for those in their 70s, and 69.8 for those in their 80s [7]. As Korea is the fastest aging country in the world [8], and psychosocial difficulties due to the high poverty rate in older adults are emerging as a serious social issue, it is estimated that the suicide problem in Korean older adults will intensify [9,10].

Compared to other age groups, older adults need special attention in that they have a particularly high risk of self-harm. While self-harm experience in older adults is often a result of a suicide attempt, elderly patients’ subsequent self-injurious behavior often leads to completed suicide, with increased mortality from medical complications and heightened vulnerability to mental distress [11,12]. In fact, the rates of mental health problems are reported to be high in older adults (approximately 15% of adults aged ≥60 years have depression) [13], and self-injurious behavior can lead to fatal life threats due to the weakening of physical functions in old age [14,15]. Additionally, older adults are often more socially isolated than younger adults, which can delay the rescue period. Even if suicide attempts do not result in completed suicide, older adults may not receive timely treatment due to financial difficulties [16]. The ratio of deliberate self-harm to suicide in older adults was estimated to be only 1–4:1 [17,18], while there were 10–20 cases of deliberate self-harm per suicide death in the whole population [19]. This difference in ratio means that older adults use more lethal methods for attempting suicide than younger adults. In a similar context, it has been reported that older people are more likely to use extreme methods, such as gunfire or pesticides, to harm themselves [20,21]. Therefore, as a preventive measure, exploring the risk factors that affect the deliberate self-harm experience of older adults is a significant task.

Various factors influence deliberate self-harm in old age. Mental disorders, such as depression, anxiety, dementia, schizophrenia, and alcoholism, are typical [22,23,24,25]. Studies report that approximately 33% to 93% of older adults are affected by depression, which may lead to self-harm [26,27,28]. Chronic diseases (including cardiovascular disease, musculoskeletal disorders, and neurological problems) and physical disabilities that affect physical functions have also been reported to increase the risk of deliberate self-harm in later life [29,30,31,32]. In addition, those who were male and older felt a greater desire to self-harm [33,34]. 

Although limited in number, several studies of Asian populations admitted to hospitals with deliberate self-harm have also contributed to the literature by identifying the characteristics of older adults with deliberate self-harm experiences. For instance, Kim and colleagues (2011) divided 388 Korean suicide attempters into two groups, under 65 years old and over 65 years old, and compared the characteristics of each group [21]. The results showed that more women under the age of 65 attempted suicide (69.8%), while more men aged >65 years attempted suicide (50.9%). In addition, only 24.5% of the group under the age of 65 reported that they had a problem with physical health, while 75.4% of the older age group did have problems with physical health. The older age group recorded a higher risk rating score as they used more lethal means of suicide attempt, such as pesticides; therefore, they suffered from a greater degree of physical damage compared to the younger age group. In contrast, the average rescue rating score in older adults was lower than that in younger adults, due to the low accessibility to rescue means. In line with *Conwell and Thompson’s (2008) study* [35], these study results suggest that self-harm attempts can lead to more fatal outcomes in older age groups than in other age groups. Studies of hospitalized suicide attempters among Chinese older adults found that the effects of long-term physical illness, delusional disorder, depressive disorder, anxiety, anger, sadness, alcohol misuse, and having no care giver were valid in predicting self-injurious behavior in later life, conforming to empirical findings of Western reports [36,37,38]. A study by Kim (2014), who conducted in-depth interviews with 35 Korean older adults who had attempted suicide, is also noteworthy [39]. As the life experiences of older adults who had previously attempted suicide were explored, the study participants were found to suffer from continued health problems, psychological distress, and high dependence on tranquilizers after their suicide attempt; as a result, they were caught in a vicious cycle of repeated self-injurious behavior in later life. 

However, studies on this age group are sparse, despite the high rate of suicide in later life and its association with self-injurious behavior. This is because, numerically, the number of self-harm attempts has previously been higher in the younger group than in older adults [22,34]. In addition, although Korea has the highest suicide rate in old age and the fastest aging population in the world, most studies on the experiences of self-harm in later life are limited to cases in the United States and Europe [32], and no attempt has yet been made to directly explore the factors affecting the deliberate self-harm experiences of Korean older adults. In the case of Kim et al.’s (2011) study, it is difficult to generalize the study results as the number of cases of older adults who experienced self-harm was limited to only 57 [21]. Moreover, previous studies have limitations in that they only tend to focus on either mental disorders or physical factors when exploring the causes of deliberate self-harm in later life. To fully understand the driving force of deliberate self-harm in older adults, it is necessary to comprehensively evaluate the effects of all potential risk factors that might lead to deliberate pain experience.

Thus, this study aims to identify the characteristics of older adults admitted to hospitals with deliberate self-harm, and analyze the risk factors affecting their self-injurious behavior. To this end, we used data from *The Korean National Hospital Discharge In-depth Injury Survey*, from 2010 to 2019, managed by the Korea Centers for Disease Control and Prevention. Based on the results, this study seeks to contribute to the theorization of self-harm experience in later life, in association with health promotion of older adults in an aging society. In addition, as several studies have raised concerns about the heightened vulnerability of older adults to suicide attempts, due to psychosocial difficulties caused by COVID-19 [40,41,42], it is estimated that the results of this study will provide empirical evidence for establishing effective intervention plans for elderly suicide attempts, regarding the pandemic situation.

## 2. Methods

### 2.1. Data

The Korea Disease Control and Prevention Agency (KDCPA) developed the Korea National Hospital Discharge In-depth Injury Survey (KNHDIS), a nationally representative survey of injury-related discharges from general hospitals in 2005, to understand the scale of injuries, identify risk factors, and provide data supporting prevention policies and intervention strategies. The KNHIDS collected data on approximately 9% of the discharged cases from medical institutions, including those of hospitals with 100 or more beds, general hospitals, and secondary community health centers. However, single specialties, long-term care hospitals, geriatric hospitals, military hospitals, and rehabilitation hospitals were excluded, even if they had 100 or more beds. Data for this study were provided after deliberation by the KDCPA, excluding variables that can be identified, such as medical institution code numbers and patient registration numbers. 

### 2.2. Methods 

This study was conducted on patients hospitalized after intentional self-harm attempts, from 2010 to 2019. Patients aged 50–100 years were included in this analysis. KNHDIS data were collected on patients’ age, gender, residence zip code, type of insurance, diagnoses, external cause codes based on the International Classification of Diseases 10th revision (herein after, ICD-10), hospital admission date, discharge date, and injury-related codes, such as the mechanism and place of injury occurrence, based on the International Classification of External Causes of Injuries. In this study, intentional self-harm inpatients were identified as cases with the main diagnosis classification of injury (ICD-10: S00-T78) and cases with extrinsic codes of an external cause (ICD-10: X60–X84 and Y10–Y34) [43,44,45,46]. Non-intentional self-harm inpatients were identified as cases with the main diagnosis classification of injury (ICD-10: S00-T78) and with an external cause classification (ICD-10: V00-X59, X85-Y09, and Y35-Y36) [47].

### 2.3. Data Variables

The independent variables were patient and clinical characteristics and treatment outcomes. Patient characteristics included gender, insurance type, and age as continuous variables. Insurance type was classified according to medical aid. Clinical characteristics, disease severity, and mental and physical diseases were recorded. Disease severity was derived from the Charlson Comorbidity Index (CCI) using the subgroup code for severe comorbidity (CCI > 3), mild comorbidity (CC1 = 1 or 2), and no reported comorbidity (CCI = 0) [48]. For mental and physical diseases associated with suicidal behavior, 21 clinical diseases were identified using the ICD-10 code [49,50]. For treatment outcomes, length of hospital stay (i.e., the period from the date of admission to discharge) and death were selected. Cases of hospitalization and discharge on the same day were considered as one, and cases with a standard deviation of three or more were considered extreme outliers and excluded from the study [51]. Death was included in the form of discharge unlikely (discharge to the end of life).

### 2.4. Data Analysis

All analyses were performed using descriptive statistics in STATA version 17.0. The analyses were divided into non-self-harm and self-harm, then chi-square test, Fisher’s exact test, and t-test were performed for patient and clinical characteristics. Logistic regression analysis was performed to calculate the odds ratio and 95% confidence interval (CI) for the association between independent and dependent variables. A chi-square test and t-test were performed to assess the treatment outcomes.

## 3. Results 

Table 1 shows the demographic characteristics and clinical diseases of older hospitalized patients (n = 123,635) by group: non-self-harm injury group (n = 120,773; 97.7%) and self-harm injury group (n = 2862; 2.3%). The non-self-harm injury group had more females (52.7%) than males, and the average age was 66.3 (±10.8) years. The patients in this group received medical aid (6.8%), and a total of 102,053 (84.5%) patients did not have a comorbidity: 13,283 (11.0%) had a CCI weighting of 1–2 and 5437 (4.5%) had a weighting of over 3. Among the clinical diseases, 1.0% had mental health conditions, 0.5% had depression, 1.5% had dementia, 0.1% had schizophrenia-related psychoses, 0.1% had bipolar disorder, 0.4% had anxiety disorders, 2.1% had neurological disorders, 0.5% had other disorders of the nervous system, 1.6% had cerebrovascular disease, 0.8% had malignancies, 0.1% had lung cancer, 0.1% had prostate cancer, 0.8% had chronic lower respiratory disease, 1.3% had liver diseases, 2.0% had arthritis/arthroses, 0.2% had alcohol misuse and dependence, and 0.0% had drug-related dependence. 

The self-harm injury group had more males (53.87%) than females, with an average age of 67.3 (±11.3) years. The patients in this group had medical aid (9.4%), and a total of 2255 (78.8%) patients did not have a comorbidity: 413 (14.4%) had a CCI weighting of 1–2 and 194 (6.8%) had a weighting of over 3. Of all the patients, 23.2% had mental health conditions, 21.2% had depression, 2.3% had dementia, 0.9% had schizophrenia-related psychoses, 0.4% had bipolar disorder, 0.4% had anxiety disorders, 3.6% had neurological disorders, 1.4% had other disorders of the nervous system, 2.2% had cerebrovascular disease, 2.1% had malignancies, 0.4% had lung cancer, 0.2% had prostate cancer, 1.2% had chronic lower respiratory disease, 2.7% had liver diseases, 0.6% had arthritis/arthroses, 3.3% had alcohol misuse and dependence, and 0.8% had drug-related dependence.

Table 2 shows the risk of self-harm injury according to the demographic characteristics and clinical diseases of older hospitalized patients. After adjusting for various factors influencing self-harm injury, women’s risk of self-harm injury was 0.687 times lower than that of men (OR = 0.687, 95% CI = 0.633–0.746). As age increased by 1 year, the risk of self-harm injury increased 1.012 times (OR = 1.012, 95% CI = 1.008–1.016). Older adults with financial difficulties, who received medical aid, had 1.168 times higher odds of self-harm hospitalization than those who did not (OR = 1.168, 95% CI = 1.013–1.348). CCI was 1.392 times higher for 1–2 points and 1.341 times higher for ≥3 points when 0 points was the standard (OR = 1.392, 95% CI = 1.157–1.676; OR = 1.341, 95% CI = 1.006–1.787).

Among the 21 clinical diseases related to self-harm injury, the odds of self-harm hospitalization in patients with mental health conditions were 10.148 times higher (OR = 10.148, 95% CI = 6.613–15.573); 6.114 times higher in those with depression (OR = 6.114, 95% CI = 4.052–9.224); 0.570 times lower in those with dementia (OR = 0.570, 95% CI = 0.412–0.789); 0.445 times lower in those with anxiety disorders (OR = 0.445, 95% CI = 0.324–0.611); 0.290 times lower in those with cerebral palsy and other paralytic syndromes (OR = 0.290, 95% CI = 0.148–0.565); 2.596 times higher in those with other disorders of the nervous system (OR = 2.596, 95% CI = 1.822–3.698); 0.670 times lower in those with diabetes (CI = 0.670, 95% CI = 0.543–0.826); 1.732 times higher in those with malignancies (OR = 1.732, 95% CI = 1.131–2.653); 0.254 times lower in those with arthritis/arthroses (CI = 0.254, 95% CI = 0.156–0.413); 8.322 times higher in those with alcohol misuse and dependence (OR = 6.216, 95% CI = 6.216–11.141); and 87.098 times higher in those with drug-related dependence (OR = 87.098, 95% CI = 37.390–202.893) in the absence of each disease.

## 4. Discussion

This study sought to identify the association of both mental disorders and physical illness with self-harm hospitalized injury over 9 years among older adults in Korea. To our knowledge, this is the largest prospective study to estimate the risk of self-harm in older Korean adults. Its key findings showed that older adults’ gender and age were associated with self-harming behavior resulting in hospitalization; that is, older male adults had higher odds of self-harm hospitalization than their female counterparts. This is in line with a previous study by Kim et al. (2011), which showed more suicide attempts among older male adults than among older female adults [21]. The results of this study provide evidence of cultural and ethnic influences on self-harming behavior by gender. Korean male older adults are more prone to traditional gender roles and patriarchal norms that highlight men’s financial responsibility [52]; thus, elderly men are more sensitive and mentally vulnerable to economic hardship in later life than their female counterparts, resulting in self-harm hospitalization. Moreover, an increase in age was associated with higher odds of hospitalization due to self-harm. Previous studies have shown similar results, where the prevalence of suicidal ideation was notably higher among elderly inpatients than among other age groups [53]. The positive association between poverty and hospitalization for self-harm is consistent with previous studies [21,54]. 

In line with previous studies, the type of mental and physical illness was significantly associated with self-harm behavior, resulting in self-harm-related hospitalization [22,23,24,25,32]. Among the self-harm and non-self-harm injury cohorts examined, this study found that self-harm hospitalized injuries were associated with higher odds of mental health conditions. A systematic review that examined factors associated with self-harm behavior among older adults revealed that psychiatric history, such as depression, malignancies, alcohol misuse and dependence, and drug-related dependence were the most commonly reported diagnoses [32,47]. The results of this study showed that some physical and mental disorders were associated with a decreased risk of self-harm-related hospitalization among older adults; those with arthritis, cerebral palsy, other paralytic syndromes, and dementia had lower odds of self-harm hospitalization than their counterparts. A possible explanation for this is that their limited physical and mental capabilities may have hindered their ability to engage in self-harm behavior. 

Findings regarding anxiety disorders are noteworthy. Studies have highlighted a long-established association between anxiety disorders and self-harm injury as it increases the odds of self-harm [32,47]. Contrary to previous findings, this study’s results showed that older adults with anxiety disorders had lower odds of self-harm hospitalization. One possible explanation for this is the issue of detection; in other words, psychiatrists may not be present in an emergency, which may limit the diagnosis of patients’ anxiety disorders. Another possible explanation is the type of data collection. The data for this study were obtained from hospitals with 100 or more beds; thus, participants admitted to psychiatric hospitals with 100 or fewer beds were not included in the analysis. Replication of these findings from patients admitted to hospitals with 100 or fewer beds is necessary before a firm conclusion about the association between anxiety disorders and self-harm hospitalization can be drawn. 

In addition, contrary to previous findings, the results from this study suggested that older adults with diabetes had lower odds of self-harm hospitalization than participants with non-self-harm injuries. This finding can be attributed to the Korean government’s collaborative efforts with community health centers and private medical centers to provide support for patients with hypertension and diabetes. For example, the Korea Disease Control and Prevention Agency (Korea CDC) provides community-based programs specifically for individuals with hypertension and diabetes. Such programs include medical subsidies for patients aged ≥65 years, education, and counseling services to help improve patients’ self-management skills regarding taking medication and maintaining physical health. This anticipated medical attention and community support for older adults with diabetes may have helped them gain a more positive attitude, alienating suicidal behavior.

## 5. Conclusions

Self-harm behavior is subtle by nature and difficult to detect; thus, the issue requires more systematic attention from researchers and clinicians. The results of this study showed that adults who are older, male, and those with financial problems, mental health conditions, depression, disorders of the nervous system, alcohol misuse and dependence, and drug-related dependence had a greater risk of performing self-harm behavior. The preliminary results from this study offer a foundation to build suicide prevention policies for older adults at risk of conducting self-harm behavior.

The findings of this study should be interpreted considering the following limitations. First, owing to the nature of self-harm injuries, it is plausible that the number of self-harm injuries may be greater than previously reported. As Mitchell and colleagues (2017) stated [47], individuals may choose not to disclose the true cause of their injuries, which could affect the interpretation of the data. Similarly, individuals with a higher socioeconomic status were less likely to report suicide attempts than those with a lower status [55]. Moreover, older adults who self-harmed and were not hospitalized because of sudden death were excluded from the study. Second, the alcohol use questionnaire was self-reported, which could have been influenced by socially desirable responses, limiting the interpretation of the data. Despite these limitations, the current study contributes to the enhanced understanding of older Korean adults by identifying risk factors associated with self-harm hospitalization. The results of this study will provide useful information to help detect risk factors that may lead to hospitalization for self-harm among older Koreans.

## Figures and Tables

**Table 1:** Demographic characteristics and clinical diseases of older hospitalized patients with non-self-harm injury and self-harm injury.

Characteristics	Non-Self-Harm Injury (n = 120,773)		Self-Harm Injury (n = 2862)		χ^2^/t	*p*
	n, Mean	%, SD	n, Mean	%, SD		
**Demographic characteristics**						
Gender						
Male	57,151	47.3	1541	53.8	47.697	0.000
Female	63,622	52.7	1321	46.2		
Age	66.3	10.8	67.3	11.3	−5.017	0.000
Medical aid						
No	112,583	93.2	2594	90.6	29.263	0.000
Yes	8190	6.8	268	9.4		
CCI *						
0	102,053	84.5	2255	78.8	72.344	0.000
1–2	13,283	11.0	413	14.4		
3+	5437	4.5	194	6.8		
**Clinical disease**						
Mental health conditions	1172	1.0	664	23.2	9400.000	0.000
Depression	623	0.5	608	21.2	12,000.000	0.000
Dementia	1760	1.5	65	2.3	12.733	0.000
Schizophrenia-related psychoses	122	0.1	27	0.9	164.814	0.000
Bipolar disorder	64	0.1	12	0.4	61.060	0.000
Anxiety disorders	505	0.4	95	3.3	487.261	0.000
Neurological disorders	2529	2.1	103	3.6	30.388	0.000
Cerebral palsy and other paralytic syndromes	719	0.6	12	0.4	1.474	0.225
Other disorders of the nervous system	603	0.5	40	1.4	43.609	0.000
Cerebrovascular disease	1938	1.6	62	2.2	5.542	0.019
Diabetes	10,584	8.8	272	9.5	1.913	0.167
Malignancies	963	0.8	59	2.1	54.498	0.000
Lung cancer	159	0.1	11	0.4	13.001	0.000
Genital cancers	76	0.1	2	0.1	0.021	0.703
Prostate cancer	86	0.1	6	0.2	7.206	0.007
Chronic lower respiratory disease	989	0.8	35	1.2	5.556	0.018
Liver diseases	1618	1.3	76	2.7	35.817	0.000
Arthritis/arthroses	2382	2.0	18	0.6	26.505	0.000
Male genital disorders	100	0.1	1	0.0	0.785	0.734
Tinnitus	118	0.1	1	0.0	1.145	0.532
Pain	1323	1.1	34	1.2	0.221	0.639
Alcohol misuse and dependence	287	0.2	94	3.3	844.768	0.000
Drug-related dependence	9	0.0	22	0.8	646.296	0.000

* CCI = Charlson Comorbidity Index.

**Table 2:** Multiple logistic regression.

Cause	OR	*p*	95% CI
Gender (Male) Female	0.687	0.000	0.633–0.746
Age	1.012	0.000	1.008–1.016
Medical aid (No) yes	1.168	0.033	1.013–1.348
CCI (0) 1–2	1.392	0.000	1.157–1.676
3+	1.341	0.045	1.006–1.787
Mental health conditions (No) Yes	10.148	0.000	6.613–15.573
Depression (No) Yes	6.114	0.000	4.052–9.224
Dementia (No) Yes	0.570	0.001	0.412–0.789
Schizophrenia-related psychoses (No) Yes	0.832	0.530	0.469–1.477
Bipolar disorder (No) Yes	0.553	0.120	0.262–1.166
Anxiety disorders (No) Yes	0.445	0.000	0.324–0.611
Neurological disorders (No) Yes	0.906	0.423	0.710–1.154
Cerebral palsy and other paralytic syndromes (No) Yes	0.290	0.000	0.148–0.565
Other disorders of the nervous system (No) Yes	2.596	0.000	1.822–3.698
Cerebrovascular disease (No) Yes	0.837	0.269	0.610–1.148
Diabetes (No) Yes	0.670	0.000	0.543–0.826
Malignancies (No) Yes	1.732	0.012	1.131–2.653
Lung cancer (No) Yes	0.775	0.540	0.343–1.750
Genital cancers (No) Yes	0.550	0.448	0.117–2.581
Prostate cancer (No) Yes	0.547	0.275	0.186–1.615
Chronic lower respiratory disease (No) Yes	0.707	0.105	0.466–1.075
Liver diseases (No) Yes	1.250	0.131	0.936–1.671
Arthritis/arthroses (No) Yes	0.254	0.000	0.156–0.413
Male genital disorders (No) Yes	0.475	0.460	0.066–3.426
Tinnitus (No) Yes	0.274	0.209	0.037–2.062
Pain (No) Yes	0.874	0.489	0.597–1.280
Alcohol misuse and dependence (No) Yes	8.322	0.000	6.216–11.141
Drug-related dependence (No) Yes	87.098	0.000	37.390–202.893

( ): reference; CCI = Charlson Comorbidity Index.

## Data Availability

The data supporting the findings of this study was obtained from Korea Centers for Disease Control and Prevention under the terms of a data use agreement which prohibits redistribution of the data. The original data sets can be obtained by any qualified researcher from https://www.kdca.go.kr/injury.
